# Erratum to Inactivation of FOXO1 induces T follicular cell polarization and involves angioimmunoblastic T cell lymphoma

**DOI:** 10.20892/j.issn.2095-3941.2019.0374

**Published:** 2020-02-15

**Authors:** Meifang Xu, Fei Wang, Hong Chen, Lin Liu, Wenwen Liu, Yinghong Yang, Qiaoling Zheng, Lihong Zhang, Xiaoxuan Li, Suxia Lin, Shengbing Zang

**Affiliations:** ^1^Department of Pathology, Fujian Medical University Union Hospital, Fuzhou 350001, China; ^2^Division of Pediatrics, University of Texas MD Anderson Cancer Center, Houston, TX 77030, USA; ^3^Department of Pathology, The First Affiliated Hospital of Fujian Medical University, Fuzhou 350004, China; ^4^Department of Pathology, Fujian Medical University, Fuzhou 350004, China; ^5^Sun Yat-sen University Cancer Center, State Key Laboratory of Oncology in South China, Collaborative Innovation Center for Cancer Medicine, Guangzhou 510060, China; ^6^Department of Pathology, Sun Yat-sen University Cancer Center, Guangzhou 510060, China

In the published article^1^, an error appeared in Acknowledgments on page 753. We omitted Natural Science Foundation of Guangdong Province from Acknowledgments, and we updated it as below. We apologize for the errors and for any confusion it may have caused.

## References

[1] Xu M, Wang F, Chen H, Liu L, Liu W, Yang Y (2019). Inactivation of FOXO1 induces T follicular cell polarization and involves angioimmunoblastic T cell lymphoma. Cancer Biol Med.

